# Online cognitive-behavioral intervention for stress among English as a second language teachers: implications for school health policy

**DOI:** 10.3389/fpsyt.2023.1140300

**Published:** 2023-11-29

**Authors:** Angela Eze, Mary O. Anyebe, Rebecca G. Nnamani, John C. Nwaogaidu, Patricia U. Mmegwa, Esther A. Akubo, Victoria N. Bako, Salome N. Ishaya, Matthew I. Eze, Francis O. Ekwueme, Ngozi N. Awokwe, Uchechukwu H. Ekwueme, Zipporah O. Omenma, Joseph N. Chukwuma, Benjamin A. Amujiri, Lucia A. Bitrus

**Affiliations:** ^1^Alex Ekwueme Federal University Ndufu Alike, Ikwo, Abakaliki, Ebonyi State, Nigeria; ^2^University of Jos, Jos, Plateau State, Nigeria; ^3^University of Nigeria, Nsukka, Nigeria; ^4^Veritas University of Abuja, Abuja, Nigeria

**Keywords:** online CBI, job-stress, English as second language, teachers, anxiety, policy

## Abstract

**Introduction:**

Stress is one of the highest-ranked work-related injuries worldwide and has become almost universal among the Nigerian workforce. English as a Second Language (ESL) teachers face enormous work-related threats that lead to occupational stress. When ESL teachers are stressed, students' language development and entire educational progress are at risk. This is mostly underscored as English, though a second language, serves as the language of instruction in Nigerian schools. As a result, managing occupational stress is particularly important for ESL teachers, as it is among the definitive ways of improving ESL learning and overall educational outcomes. This study examined the effectiveness of online cognitive behavioral intervention (o-CBI) in lowering occupational stress among ESL teachers.

**Method:**

ESL teachers with at least 1 year of experience were among the participants (*N* = 89). Participants were divided into two groups: the intervention group (*N* = 44) and the control group (*N* = 45). For 9 weeks, the experimental group engaged in nine sessions of 2 h of the o-CBI program. The Single Item Stress Questionnaire (SISQ), the Satisfaction with Therapy and Therapist Scale-Revised (STTS–R), and the Teachers' Stress Inventory (TSI) were the measures used to collect primary and secondary data. Four sets of data were collected at baseline, post-test, and follow-up 1 and 2 evaluations. The data were analyzed using mean, standard deviation, *t*-test statistics, repeated measures ANOVA, and bar charts.

**Results and discussion:**

Compared to the control group, the o-CBT group had significantly lower TSI scores at the post-test (Time 2) and follow-up evaluations (Times 3 and 4). Between pre-, post-, and follow-up 1 and 2 measurements, there were no significant differences in occupational stress index scores in the control group. It was concluded that o-CBI is effective in job-stress treatment among ESL teachers. In addition, implications for school health policy are discussed. The o-CBI for occupational stress was well received by the participants, showing high acceptability among ESL teachers.

## Introduction

Work-related stress constitutes approximately 85% of psychosocial workplace risky exposures (1, 2), and chronic stress accounts for increased work-related injuries that endanger workers' health and wellbeing (3–5). Normally, employees become overwhelmed by work-related stress (6) due to a mismatch in time, resources, and work demands from colleagues, bosses, or other structures of the organization (2, 7). Psychological stress indicates exposure to intense traumatic experiences that can cause anxiety, anger, and sadness in the workplace (8). Stress associated with the occupation is a common issue, affecting approximately 88% of teachers worldwide (9–12). Compared to other employees in other occupations, teachers are the most stressed (13–15), and language teaching tends to add more complications that cause increased stress among teachers (16, 17).

In education, stress is defined as a frustrating, emotional experience caused by the process of teaching/learning management and is demonstrated in some emotional conditions, including anger, strain, anxiety/tension, frustration, or depression (18, 19). Stress is a chronic negative experience that endangers educators' self-confidence and wellbeing and jeopardizes the educational process (20). Teaching English as a second language (TESL) is even more challenging, and ESL teachers are twice more likely to be stressed than other language teachers and about three times more vulnerable to job stress than other employees (21–23). Teaching English as a second language necessitates high English language skills as well as pedagogical skills, which are most often lacking in teachers who often struggle with English because they are not native English speakers (19, 23). ESL teachers may experience heightened stress because of the unique need for appropriately modified training, which can be stressful for instructors (14, 20).

Consequently, teachers of ESL tend to be more vulnerable to increased stress due to reduced professional resources (24–26), poor professional development (27), and poor salaries (28). A survey study (25) found that 80% of ESL teachers in Namibia lack teaching materials and resources that create the learning environment. Nigerian-based studies (29, 30) found similar challenges, exacerbated by little or no contact with first-language English speakers and with no background knowledge to enhance communication in English (31). Such conditions tend to increase teachers' responsibility to build learners' communication skills in English, thereby increasing teachers' job stress (19, 25).

Job stress among ESL teachers has negative implications for their psychosocial wellbeing, the students they teach, and the entire education system (20, 23). Extant literature suggests that workplace stress tends to undermine/or weaken teachers' job performance (32–36). Stress may lead to increased depression cases among teachers (23), which has far-reaching effects on school health outcomes (37).

Similar to the findings in other countries, high stress levels have consistently been the outcome of research on ESL teachers in Nigeria (30). However, studies in the Nigerian context with regard to stress among ESL teachers are very scarce. The few studies available on stress among ESL teachers in Nigeria were survey studies with large samples, suggesting that stress is widespread (29). While most of such studies present similar narratives regarding ESL teachers' stress, not many Nigerian studies have delved into stress management among the population. This suggests the need for stress management intervention among ESL teachers (16, 38). With the increasing need for building students' skills in the use of English in Nigeria (21, 30), there is an urgent requirement for supporting teachers in obtaining the maximum output on students' outcomes. In this randomized trial, we employed an online cognitive-behavioral intervention to reduce occupational stress among ESL teachers in Plateau State, Nigeria. This study is expected to benefit both the ESL teachers, the school, and the students, as it will ultimately improve school social, health, and academic outcomes, especially as English is used as a medium of instruction in Nigeria.

## Job stress

Stress arises if there is a conflict between external demands/events and the individual's capacity to deal with such events or activities (39). There are three major dimensions or models in which stress might be experienced: the stimulus-based model, the response-based model, and the cognitive transactional process model (39, 40). The stimulus-based perspective is of the view that stress occurs when there are objectively activating events called stressors (40). According to this model, stress level is simply a measure of stress sources or stressful events/experiences.

From this perspective, the response-based model is based on the stance that stress is the emotional, physiological, and behavioral reaction/response to the objective stressor. In this regard, an individual's stress level is a combination of stress sources and subjective personal factors/modifiers, such as their interpretation/cognition perspective on the stressor. According to the transaction-based model, stress is caused by negative sensory interpretations/cognitions about the triggering condition (39, 41, 42). This model holds that stress is the subjective feeling of emotional, behavioral, and physical symptoms that are consequential to the interaction between the stimulus and the response (39, 42). In this study, we followed an encompassing approach to define stress as both the process and product of the interaction between stressors, the perception of stressors, and the symptomatic outcomes following the interaction between stressors and the outlook. Based on this eclectic viewpoint, we define job stress, which is our study variable.

Job stress is described as a negative subjective emotion that occurs when a worker's perceived personal and social skills to suitably deal with work demands exceed the needs of the job (43). It is a typical event in the lives of teachers, but it turns negative when they become chronically activated as a result of prolonged exposure to stressors and a dysfunctional attitude toward their work. This results in major consequences for cognitive, bodily, and/or emotional health (44). Job stress can also be explained as damaging bodily and psycho-emotional reactions that follow when an employee lacks the capabilities and resources to cope with their work requirements (12). Operationally, job stress refers to the psycho-physiological condition where the perceived work-related demands become so overpowering that they have negative effects on employees' emotional and physical health. Thus, job stress includes both the perceived stress sources associated with a job and the symptomatic reactions due to such sources.

Job stress is generally caused by working conditions (e.g., workover/underload, long hours, too many decisions, deadlines, and time pressures); organizational roles (e.g., role ambiguity, role conflict, responsibility for people and things, lack of participation in decision-making, lack of support, and poor standards of performance); poor interpersonal relationships at work (e.g., workover/underload, long hours, too many decisions, deadlines, and time pressures); and poor career development (feelings of getting stagnated, under/over promotion, lack of job security, and fear of redundancy).

Employees' productivity, physical health, and overall wellbeing are jeopardized by increasing occupational stress (34, 44–46). Job stress leads to both physical and mental health symptoms (47, 48), including posttraumatic stress disorders, sleep disorders, depression, and anxiety (49). It is also implicated in fatigue/burnout (10, 50, 51), absenteeism, inefficiency, attrition (39, 52), and suicidal attempts (53). Physical symptoms such as headaches, decreased immunological function, increased musculoskeletal pain, and cardiovascular disorders have also been linked to occupational stress (54). Thus, in this study, job stress is addressed as a measure of both perceived sources of stress and their symptomatic manifestations.

### The Nigerian context regarding the development of English as a second language

Learning English as a second language is challenging and requires complex skills (55). In Nigeria, learning English as a second language has become vital and forms the basis for the entire education system. At the basic education level in Nigeria, two main languages are used in class instruction: the mother tongue and the English language (56, 57). While the mother tongue is used for teaching at the junior basic education level, the English language is used from the senior basic education level through the subsequent levels of education (58). Though a second language, the English language is also increasingly used as the official language in Nigeria (57), making it imperative in the education system. Thus, ESL is an integral part of the Nigerian educational system from the primary to the tertiary levels (57, 59).

Despite English being the second language in the Nigerian education system, the teaching and learning process faces challenges due to a shortage of qualified English teachers (55), inadequate training for teachers, and poor subject delivery (60–62). However, ESL students may present additional challenges, including those associated with grammar, pronunciation, vocabulary, slang, colloquialisms, and sentence structure (63). Hence, L2 teachers experience great difficulty in making students understand the teaching contents in English (64) and making mindful efforts to build reading and writing proficiency. Consequently, teachers who teach ESL in Nigeria are presented with high levels of demands that can leave them overwhelmed and highly stressed, which requires intervention.

### Online cognitive behavioral intervention

**Figure 1:**
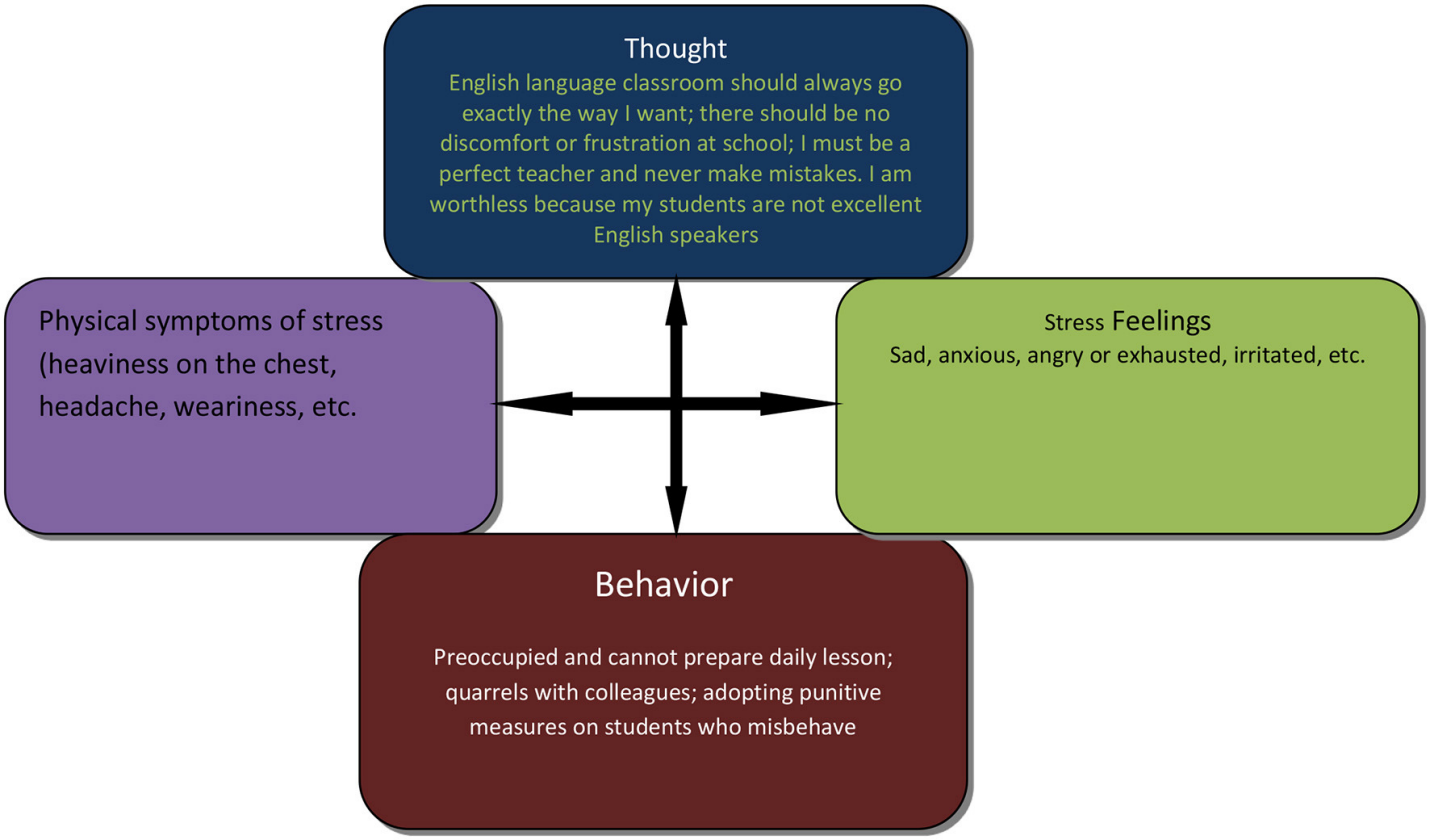
CBT formulation model of EFL teachers' stress.

The online cognitive behavioral intervention (o-CBI) is based on the cognitive behavioral therapy (CBT) developed by Beck (65). According to Beck's cognitive model, people's perceptions of events (cognition/thought) influence their feelings and behaviors (see Figure 1). What people feel about an event is a product of the way in which they interpret the event itself (65). The intervention model conceptualizes cognition at three levels: core beliefs, dysfunctional assumptions, and negative automatic thoughts (66). Core beliefs are schemas, which are deeply constructed beliefs about oneself, others, and the world. Dysfunctional assumptions are inflexible conditions that people adopt for living, while negative automatic thoughts (NATs) are involuntarily triggered thoughts about certain situations (66). Cognitive behavioral intervention (CBI) teaches clients to become their own therapists by assisting them in making sense of their current thoughts and behavioral patterns and helping them develop the skills needed to change dysfunctional patterns.

Different intervention techniques based on scientific evidence and practical efficacy have been used under cognitive behavioral therapy (CBT) to reduce stress among teachers (67–69). Internet-based stress-reducing CB intervention has been found to have small to moderate effect sizes compared to controls in both meta-analyses (70) and control trials (71, 72). It is not clear how o-CBI can be effective in reaching rural populations. Further investigation into the effect of o-CBI on ESL teachers reporting elevated stress and suffering from stress-related disorders is necessary to ensure the development of a high-quality policy structure for school mental health. Online CB interventions are delivered using electronic media in CBT modalities. Online CBI for stress is a technology-based intervention framework for reducing stress (73). It is meant to draw from occupational experience the skills necessary to develop resilience for managing stress and associated hazards in workplaces (74).

The o-CBI is based on the conviction that health-related reactions to job experiences (including stress) arise following a dysfunctional belief that accrues from work challenges (75). CBT uses psychological tools to argue and attack flawed feelings, fuzzy cognitive dispositions, and dysfunctional behaviors toward work and work experiences and to improve the professional wellbeing of workers (76). Furthermore, given the complex nature of stress-related health challenges and the time and financial cost of face-to-face group intervention, an online intervention approach was recommended for stress management (71, 73, 77–79). In the o-CBI context, online modules of CBT are implemented as either individualized self-help modalities or as a guided program based on either individual or group needs (73, 74).

Compared to traditional face-to-face therapy, online cognitive behavioral interventions could provide participants with more flexibility regarding when and where they can access the support program (80). In an undefined setting, it lowers the cost of therapeutic visits and improves support-seeking abilities (81). In this trial, o-CBI was given as a group therapy through videoconferencing, WhatsApp chats, and emails. The online component of treatment is made available in mobile apps to allow clients to participate in therapy and complete homework projects by themselves. Online therapy, according to Ong et al. (82), has the advantages of connectivity, reduced prices, and a relatively low time commitment for both therapists and clients; it helps clients take a more active role in treatment, maintains their motivation and momentum, and facilitates the exchange of information and images between participants and therapists to monitor client progress and improve transparency.

### The current study

While ESL teachers report heightened stress, no study has used online CBI programs to help them reduce job stress. Most of the related literature studies were delivered by populations other than ESL teachers. No study in the literature uses the o-CBI to reduce stress among ESL teachers. In addition, there is a lack of empirical information regarding whether ESL teachers would be satisfied with o-CBI. The current study offers insights into using o-CBI for stress reduction in ESL teachers. In addition, participants' satisfaction with o-CBI was explored.

### Research questions and hypotheses

The study was guided by the following questions: (i) Will o-CBI lead to a significant reduction in stress among ESL teachers? (ii) Will changes in job stress brought about by exposure to o-CBI among teachers be sustained over follow-up time? We, therefore, hypothesize that o-CBI would lead to a significant reduction in job stress among ESL teachers. We further claim that the o-CBI group will lead to a sustained and significant decrease in job stress in the experimental group over those in the control group.

## Materials and methods

Ethical considerations: The Faculty of Educational Research Committee, a university in Nigeria, provided ethical permission for this study. The study was also registered prospectively in the American Economic Association's registry for randomized control trials, with ID AEARCTR-0005471. The study also met the American Psychological Association's (APA) and World Medical Association's (WMA) research ethical standards (83, 84). The study participants also gave their informed written consent.

### Measures

#### The single item stress questionnaire

One of the inclusion/exclusion criteria for the study was this single-item measure of stress symptoms. In stress studies, the instrument has repeatedly been confirmed to be valid and trustworthy (85, 86), showing Chronbach's alpha reliability indices ranging from 0.80 to 0.86. The instrument uses the question, “Stress means a situation when a person feels tense, restless, nervous, anxious, or unable to sleep at night because his or her mind is troubled all the time. Do you feel that kind of stress these days?” The SISQ is scored on a 5-point scale, with 1 being “not at all” and 5 being “very.” In this study, low stress was assigned a score of 1–2, moderate stress was assigned a score of 3, and high stress was assigned a score of 4–5. For SISQ, the researcher discovered a Cronbach's alpha reliability index of 0.79 among 20 adult Nigerian workers.

#### The teachers' stress inventory

This is a questionnaire used by teachers to assess their stress levels. The TSI (87) is a 49-item questionnaire with a four-point Likert scale that was employed in this investigation. The instrument has 10 subscales that cover two major stress dimensions: stress sources (SS) and stress manifestations (SM). The SS subscale measures sources of stress and management practices, while the SM subscale measures physiological and psychological symptoms of stress (87). The TSI by Fimian (87) is quite old but has remained the best scale for measuring teachers' stress across studies (1). Owing to its unbeatable validity, TSI has been widely used in world-standard contemporary studies (88). In South Africa, the TSI was discovered to have good psychometric properties (89, 90). We also conducted a reliability test to determine the instrument's suitability in Nigeria. In this regard, data from 47 Nigerian instructors were subjected to the Cronbach alpha statistic, which resulted in a good reliability coefficient (=0.81).

#### The satisfaction with therapy and therapist scale-revised

The participants' satisfaction with the o-CBI was assessed using the STTS–R for group psychotherapy created by Oei and Shuttlewood (91). The STTS–R is a 5-point Likert scale with Strongly Disagree (1), Disagree (2), Neutral (3), Agree (4), and Strongly Agree (5) options (6). The measure consists of 13 items that address client satisfaction with the therapy, therapist satisfaction, and overall change in the client's condition. The STTS–R has excellent psychometric properties (91). Though the scale is relatively old, it has also proven valid in more recent studies (92) and is continually valuable in measuring the acceptability of internet therapy (93) and other recent clinical studies (94). The STTS–R was trial tested on 47 Nigerian teachers to validate its applicability in the Nigerian setting. Cronbach's alpha statistics yielded an alpha coefficient of 0.67, indicating that the instrument was trustworthy among Nigerian teachers.

### Participants

The study included 89 ESL teachers (men = 29; women = 60) who participated in the study. Table 1 contains detailed demographic information about the individuals. The following criteria were used to choose the participants: (a) the participant must have a stress score of 3–5 on the single-item measure of stress symptoms, indicating a moderate to high level of stress; (b) the teacher must be an English language teacher in primary or secondary schools; (c) the participant must have a personal smartphone with a functional email address and must be connected to WhatsApp; and (d) the participant must be willing to submit personal contacts and phone numbers.

**Table 1:** Summary of the cognitive behavioral therapy session content.

**Week/ module**	**Session topic**	**Details**	**Therapist's role**	**Strategies**
1	Psychoeducation	Exploring the concept of stress, its characteristics, sources, responses, and consequences. Understanding stress models and organization of the situations where participants feel anxious or stressed	- Guide the clients to recognize how stress influences their lives - Support the participants in identifying some stressors in teaching English as a second language - Guide the clients to trace some situations that get them stressed and explain them in the models of stress	Problem formulation/ identification; goal setting
2	Stress management	Understanding participants' psychological state when they feel stressed. Dealing with stressors.	- Use dialogue to probe into clients' experiences regarding stress associated with teaching the English language - Facilitate discussion of alternative ways of minimizing stressors through problem-solving - Help clients find out how their bodies react to stress, how to spot when they are stressed, and what to do to counter the emotional trigger	Conversation, problem-solving, rational coping statements
3	Relaxation	Diaphragmatic breathing and thematic imagination	- Help the clients practice relaxation techniques through deep breathing - Discuss how deep breathing can help reduce negative emotions - Lead clients to establish the link between thought and emotion	Relaxation method; hypnosis; guided visualization; reasoning tactics
4	Cognitive restructuring	Cognitive distortion: identifying thought patterns that stir up stressed emotions and considering alternative thoughts	- Lead participants to identify their own negative thoughts and belief patterns that relate to teaching ESL and state alternative thoughts and explanations	Disputation and cognitive reorganization
5	Cognitive restructuring	Treating irrational beliefs that lead to stress in teaching ESL teachers	- Guide clients to counter irrational belief patterns through disputation - Assist clients in building a positive outlook	Dissension, homework assignments; Unconditional self-acceptance;
6	Alternative thought control Techniques	Self-instructional training and time organization.	- Guide clients to discover alternative thinking patterns to help their mental health - Share information on self-talk and emotion regulation	Homework assignments, relaxation, decision-making
7	Social skills training and support	Clarifying social support resources in participants' daily lives. Assertiveness, basic assertive rights, saying no, and how to request a change of behavior	- Facilitate discussion of access to social support resources such as assertiveness, unconditional self, and others' acceptance. Building an effective social-language environment	Unconditional others and self-acceptance;
8	Emotional Self-regulation	Sharing what has been learned from the intervention and how it will be used in the future. Sharing personal issues and experiences relating to staying healthy at work and its benefits.	- Encourage participants to talk about what they have learned from the intervention and how they will use it in the future. - Support sharing of personal issues and experiences relating to staying healthy at work. Facilitate discussion of the relationship between anger and stress	Meditation; decision-making; conflict resolution
9	Humor's benefits and summary	Maintaining a good mood and optimism/relapse prevention	- Guide a positive discussion about optimism - Prevent relapse by discussing applications and techniques	Humor and irony

**Figure 2:**
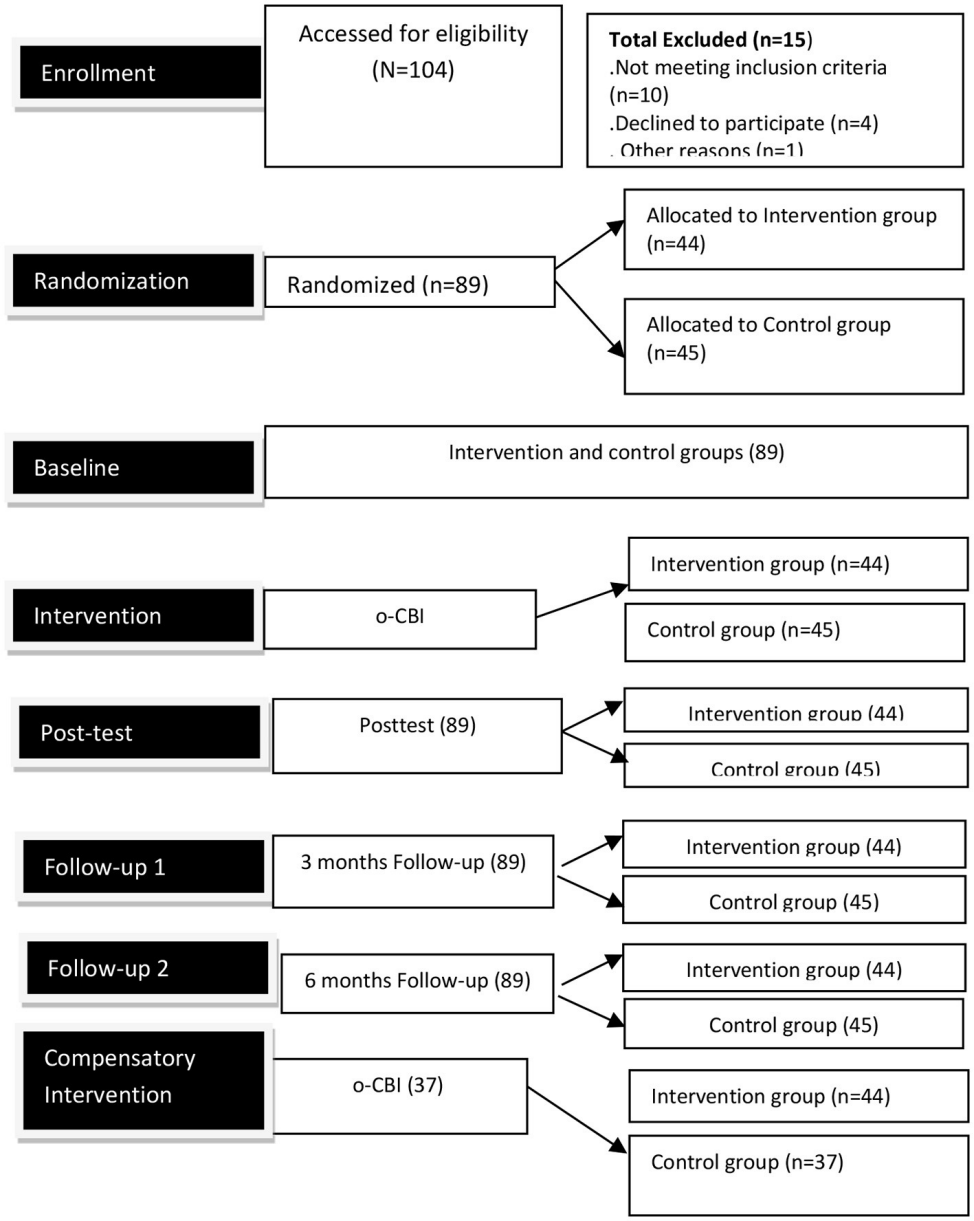
Design/participants' flow chart.

In the first stage, information about the intervention was widely shared through WhatsApp, Facebook, calls, emails, and SMS. While disseminating the information regarding the intervention, fliers and a link to the WhatsApp group were shared so that interested potential participants could join the group. A total of 89 of 104 teachers who volunteered to participate in the study were included. The 104 potential participants were evaluated for eligibility using the previously mentioned eligibility criteria. As a result, 15 potential participants were ruled out for failing to meet the inclusion criteria or for other reasons, and the 89 participants who met the criteria were included to participate (see Figure 2).

Accordingly, during stage two, the 89 teacher participants were randomly assigned to the intervention group (44 participants) or the no-intervention control group (45 participants) (see Table 1). Until the intervention was assigned, the participants were kept in the dark about the randomization method. For better communication about the stages of the intervention, the researcher created two more WhatsApp chat groups (one each for the intervention and the no-intervention control groups) and added the participants accordingly. Stage three involved baseline data collection, where both the intervention and no-intervention control groups were given a pretest utilizing TSI and SRS-18 to determine the baseline (Time 1) data. All data collected at the baseline were compared with the post- and follow-up data.

During stage four, the participants in the intervention group were exposed to o-CBI for a total of nine sessions (see Table 2), while those in the control group were waitlisted and received the intervention after the post-treatment evaluation only on individual request. For the intervention group, the o-CBI module was shared in the group after every 7 days. The participants were made to undergo the sessions alone and completed a practice activity after each session. Each participant was able to access the sessions at their own pace, provided that each session was completed, and all worksheets were completed and submitted to the researchers via email within a week before another session was shared. During the sessions, the therapists could take personalized calls, emails, and private chats to respond to the participants' queries.

**Table 2:** Participants' demographic information.

**Characteristics**		**o-CBI N (%)**	**Control N (%)**	**Total N (%)**
**Gender**				
	Men	15 (16.85)	14 (15.73)	29 (32.58)
	Women	29 (32.58)	31 (34.83)	60 (67.42)
	Total	44 (49.44)	45 (50.56)	89 (100.00)
Age	Mean	31.02	33.31	32.17
**Years of experience**				
	1–2	11 (12.35)	14 (15.73)	25 (28.08)
	3–5	19 (21.35)	17 (19.10)	36 (40.45)
	5 and above	14 (15.73)	14 (15.73)	28 (31.47)
	Total	44(49.44)	45 (50.56)	89 (100.00)
**Level of school**				
	Secondary Schools	19 (21.35)	19 (21.35)	38 (42.70)
	Primary schools	25 (28.09)	26 (29.21)	51 (57.30)
	Total	44 (49.44)	45 (50.56)	89 (100.00)
**Teachers' qualification**				
	NCE	25 (28.09)	28 (31.46)	53 (59.55)
	Bachelors' degree	18 (20.22)	17 (19.10)	35 (39.32)
	Masters' degree	1 (1.12)	0 (0)	1 (1.14)
	Total	44 (49.44)	45 (50.56)	89 (100.00)

During stage five, post-test (time 2) data were collected from both the intervention and the control groups. This occurred 2 weeks following the final intervention session. Furthermore, 3 and 6 months following the post-test evaluation, follow-up online interactions and data collection (Time 3 and 4) were conducted. Finally, the intervention program for the interested waitlisted group began immediately after the 6-month follow-up examination. The technique was the same as that for the intervention group. One of the researchers, along with four research assistants, conducted and facilitated the intervention (two experts in CBT and two occupational therapists-PhD students who were conversant with online interventions). All of the research assistants were remunerated for their study. To ensure active involvement in the intervention sessions, the researcher sent reminder messages to the participants via the WhatsApp app 1 day before the module completion deadline elapsed. The researcher was also available to answer specific questions and demands of the participants via WhatsApp and phone conversations. All the assessments were completed by completing the module in soft copies and emailing it. The intervention sessions and evaluations were conducted entirely in English. During the analysis, each assessment data point from the intervention group was compared to the data collected from the control group.

### Intervention

The o-CBI intervention comprises nine sessions of weekly online modules of CBT for stress, developed by researchers from earlier studies (66, 73, 74). The intervention was anchored on using cognitive and behavioral strategies (66) to address ESL teachers' stress by linking them to the explanatory models of stress and stressful experiences in teaching ESL. Based on the perspective used in this study, teachers' high levels of stress reactivity could be linked to their faulty perspective of their experiences rather than a direct result of their job pressures (6, 95). To this end, the negative cognitive judgment of job experiences may cause negative emotional and physical reactions (stress), limiting occupational outcomes (75). Changing perceptions toward the job through the cognitive behavioral intervention helped manage the negative reactions associated with job stressors. The o-CBI stress management intervention for ESL teachers is complete without face-to-face therapeutic contact or support but was delivered through an e-learning system, where group intervention meetings were held through videoconferencing and continuous sharing of ideas and materials via WhatsApp and emails.

The program contents are shown in Table 1. The total of nine sessions of the o-CBI program involve tasks relating to psycho-educational stress management and cognitive behavioral therapy. The intervention was planned to develop three basic stress-coping skills: problem-solving, relaxation, and emotion regulation (96). It further involves nine modules that participants work on while engaging in a weekly meeting schedule. Each module is made up of general information, audio and video files, interactive exercises, and worksheets in downloadable format, which participants complete and share with therapists. In addition, participants accessed information on stress-related topics, including time management, worrying, rumination, feelings of psychological indifference to work, sleeping habits, nutrition, social support, and exercise (96).

Participants underwent the o-CBI sessions online from the comfort of anywhere and could complete assignments within the stipulated period.

In the first two sessions, participants were exposed to an understanding of the models of stress coping mechanisms through psychoeducation and cognitive and behavioral techniques (66, 73). All sessions were held online through Zoom videoconferencing. Modules and activity materials, such as audio and video files, interactive exercises, and worksheets, were shared through email 48 h prior to each session to enable the participants to take the time to engage with the materials before the group interactions. Moreover, the therapist's line was open 24 h a day for individual therapeutic relationships. Though the weekly therapy sessions were held as group sessions, clients could contact the therapists for questions and clarifications via WhatsApp messages, calls, and emails on an individual basis. After each session, the participants were asked to complete all the worksheets in the form of homework assignments, which were submitted at any time before the subsequent session. Participants used the program both synchronously and asynchronously, as it was possible for individuals to access materials at any time, yet they were expected to participate in group meetings each week. Thus, the therapist played the role of a facilitator during the intervention period. Subsequent sessions involved cognitive restructuring and adopting functional, rather than irrational, thought styles, as summarized in Table 1.

#### Recruitment, response rates, dropouts, and adherence

We obtained informed approval from the potential participants before recruiting. A total of 104 people responded to the request to participate in the study; however, only 89 of them were accepted. Others were turned away because their qualifications did not fulfill the standards. All the 89 participants strictly adhered to the study protocol. The 44 participants in the intervention group completed all the intervention sessions and assignments. However, out of the 45 participants in the waitlisted group, only 20 indicated interest and were exposed to the compensatory intervention thereafter. As a result, there was a high percentage of adherence to this trial. In general, the participants replied quickly to online interactions, with only a few exceptions. Homework assignments were duly completed.

### Design and data analyses

With pretest, post-test, and follow-up evaluations, the current investigation used a group-randomized waitlist control trial design (97). The researcher used this design to evaluate the impact of the o-CBI on the occupational stress of ESL teachers. There were two groups of participants: the intervention (o-CBI) and control (waitlisted) groups. Demographic data were presented in numbers and percentages. The baseline data were analyzed using *t*-test statistics. To compare baseline, post-intervention, and follow-up 1 and 2 data, a repeated measures ANOVA was employed. The effect size of the intervention on the dependent measure was estimated using partial Eta square analysis. *Post-hoc* analysis was employed to ascertain the group × Time differences in the TSI scores of participants. The percentage was used to analyze the data collected through STTS–R to assess how satisfied the participants were with their therapy.

## Results

The participants' personal demographics are presented in Table 1. Men made up 32.58% of the participants, while women made up 60 (67.42%). The o-CBI group consisted of 15 (16.85%) men and 29 (32.58%) women, while the control group consisted of 14 (15.73 percent) male teachers and 31 (34.83 percent) female teachers.

In addition, within the o-CBI groups, 11 participants (12.35%) had 1–2 years of experience, and 14 participants (15.73%) had 3–5 years of experience. Similarly, within the control group, 14 participants (15.73%) had 1–2 years of experience, 17 participants (19.10%) had 3–5 years of experience, and 14 participants (15.73%) had above 5 years of experience. For the o-CBI and the control group, the average age of the participants was 31.02 and 33.31, respectively. A total of 51 (57.30%) participants were teachers in elementary schools, while 38 (42.70%) were teachers in secondary schools. The o-CBI group included 25 (28.09%) primary school teachers and 19 (21.35%) secondary school teachers, while the control group included 26 (29.21%) and 19 (21.35%) primary and secondary schools, respectively. In terms of qualifications, 25 (28.09%) and 28 (31.46%) participants had NCE in the o-CBI and the control groups, respectively; 18 (20.22%) and 17 (19.10%) participants had bachelor's degrees in the o-CBI and the control groups, respectively; and 1 (1.12%) and 0 (0%) participants had masters' degrees and above in the o-CBI and the control groups, respectively.

Table 3 displays the *t*-test statistics of the participants based on TSI subscales (SS, SM, and TTSIS) at the different evaluation times. At Time 1, the mean stress sources (SS) score of the experimental (o-CBI) group and the control group (waitlisted) were non-significantly different (t = 2.05, *p* = 0.693; η^2^_p_ = 0.001). This indicates that both the o-CBI and control groups had similar sources of job-demand perception (o-CBI group = 3.59; 0.36; control = 3.42, 39). The stress manifestation (SM) ratings of the intervention group and the control group did not also differ significantly at baseline (t = 2.28, *p* = 0.520; η^2^_p_ = 0.02). The total TSI rating of participants in the o-CBI and control groups did not differ substantially between the o-CBI group and the control group (t = 2.16, *p* = 0.492; η^2^_p_ = 0.001). This difference suggests that the participants in both groups not only perceived their employment as demanding but also had stress-related symptoms, according to the mean ratings of the two groups. The minimal η^2^_p_ in each case further strengthened the lack of a score difference based on the group.

**Table 3:** *T*-test analysis of the baseline data on participants' TSI dimensions (SS, SM, and TSI).

	**Group**	**Subscale**	**N**	**$\overset{–}{X}$, SD**	**Mean difference**	**T**	**P**	**95%CI**	**η^2^_p_**
Baseline (Time 1)	o-CBI	SS	44	3.54, 0.42	0.16	1.80	0.693	−0.01, 0.34	0.001
	Control		45	3.38, 0.42					
	o-CBI	SM	44	3.50, 0.51	0.01	2.28	0.520	0.03, 0.54	0.02
	Control		45	3.49, 0.66					
	o-CBI	TTSIS	44	3.52, 0.45	0.11	2.16	0.492	0.01, 0.43	0.00
	Control		45	3.41, 0.51					
Post-test (Time 2)	o-CBI		44	1.9, 0.96	−1.69	106.69	0.000	1.68, 3.83	0.56
	Control	SS	45	3.59, 0.43					
	o-CBI		44	1.30, 0.28	−2.26	233.03	0.000	1.28, 3.67	0.88
	Control	SM	45	3.56, 0.46					
	o-CBI		44	1.65, 0.52	−1.93	327.02	0.000	1.51, 3.72	0.79
	Control	TTSIS	45	3.58, 0.44					
Follow-up 1 (Time 3)	o-CBI		44	1.97, 1.11	−1.61	77.22	0.000	1.70, 3.86	0.47
	Control	SS	45	3.58, 0.43					
	o-CBI		44	1.59, 0.34	−1.97	478.89	0.000	1.47, 3.68	0.85
	Control	SM	45	3.56, 0.46					
	o-CBI		44	1.78, 0.63	−1.79	224.60	0.000	1.61, 3.75	0.72
	Control	TTSIS	45	3.57, 0.44					
Follow-up 2 (Time 4)	o-CBI		44	1.74, 0.58	−1.87	291.74	0.000	1.59, 3.76	0.77
	Control	SS	45	3.61, 0.38					
	o-CBI		44	1.98, 0.55	−1.96	180.94	0.000	1.82, 3.70	0.68
	Control	SM	45	3.94, 0.49					
	o-CBI		44	1.86, 0.56	−1.71	240.00	0.000	1.71, 3.73	0.71
	Control	TTSIS	45	3.57, 0.43					

SS, Stress Sources; SM, Stress Manifestation; TTSIS, Total Teachers' Stress Inventory Score;$\overset{–}{X}$, Mean; SD, Standard Deviation; df, Degree of Freedom, t, *t*-test statistic; p, probability value; CI, Confidence Interval; η^2^_*p* =_ partial eta square/effect size.

Table 3 further indicates the scores for SS, SM, and TTSIS of both the o-CBI and control groups during the post-test, follow-up 1, and follow-up 2 assessments. At Time 2, 3, and 4 (post-treatment) evaluations, the data revealed significant effects of o-CBI on all the stress dimensions (SS, SM, and TTSI). The participants in the o-CBI group had a substantially lower mean score in the SS subscale compared to the control group at Time 2 (*p* = 0.000, η^2^_p_ = 0.56); Time 3 (*p* = 0.000, η^2^_p_ = 0.47); and Time 4 (*p* = 0.000, η^2^_p_ = 0.77). Further, the mean rating of the o-CBI group for stress manifestation (SM) decreased significantly as compared to the control groups at Time 2 (*p* = 0.000, η^2^_p_ = 0.88); Time 3 (*p* = 0.000, η^2^_p_ = 0.85); and Time 4 (*p* = 0.000, η^2^_p_ = 0.68). This consistent reduction in the SM score for the o-CBI group indicates that the reduction in stress manifestation was maintained. This suggests that o-CBI could lower individuals' negative perceptions of work-related stress and mitigate their stress symptoms. The total stress score (TTSIS) of the o-CBI group was significantly lower than that of the control group at the post-test (Time 2) (*p* = 0.000, η^2^_*p*_ = 0.79); follow-up 1 (Time 3) (*p* = 0.000, 2 = 0.72), and follow up 2 (Time 4) (*p* = 0.000, η^2^_*p*_ = 0.71). These primary outcomes provided a detailed description of how o-CBT deflated teachers' stress both in part (both SS and SM dimensions) and globally (TTSIS) from pre- to post-intervention and follow-up tests.

A simple main effect analysis was performed using repeated measures ANOVA only for the total TSI score (TTSIS) and is presented in Table 4. The data in Table 4 further shows that the global stress scores of the o-CBT and control groups did not vary significantly at Time 1—before the intervention (*p* > 0.001) but varied significantly at the post-test (Time 2), follow-up 1 (Time 3), and follow-up 2 (Time 4), with a *p*-value of < 0.001 for each case.

**Table 4:** Repeated measure ANOVA for the simple main effects of the o-CBT intervention on the participants' stress scores.

**Time**	**Group**	**N**	**Mean**	**SD**	**Sum of squares**	**Df**	**Mean square**	**F**	**P**
Time 1	Control	44	3.52	0.47	0.87	1	0.87	4.57	0.04
	o-CBI	45	3.41	0.40					
Time 2	Control	44	3.58	0.44	69.03	1	69.03	326.68	< 0.001
	o-CBI	45	1.65	0.52					
Time 3	Control	44	3.57	0.44	64.75	1	64.75	221.86	< 0.001
	o-CBI	45	1.78	0.63					
Time 4	Control	44	3.57	0.43	62.44	1	62.44	303.69	< 0.001
	o-CBI	45	1.86	0.56					

Type III Sum of Squares. N, number in group; SD, standard deviation; Df, Degree of Freedom; p, probability value; η^2^p = partial eta square/effect size.

**Figure 3:**
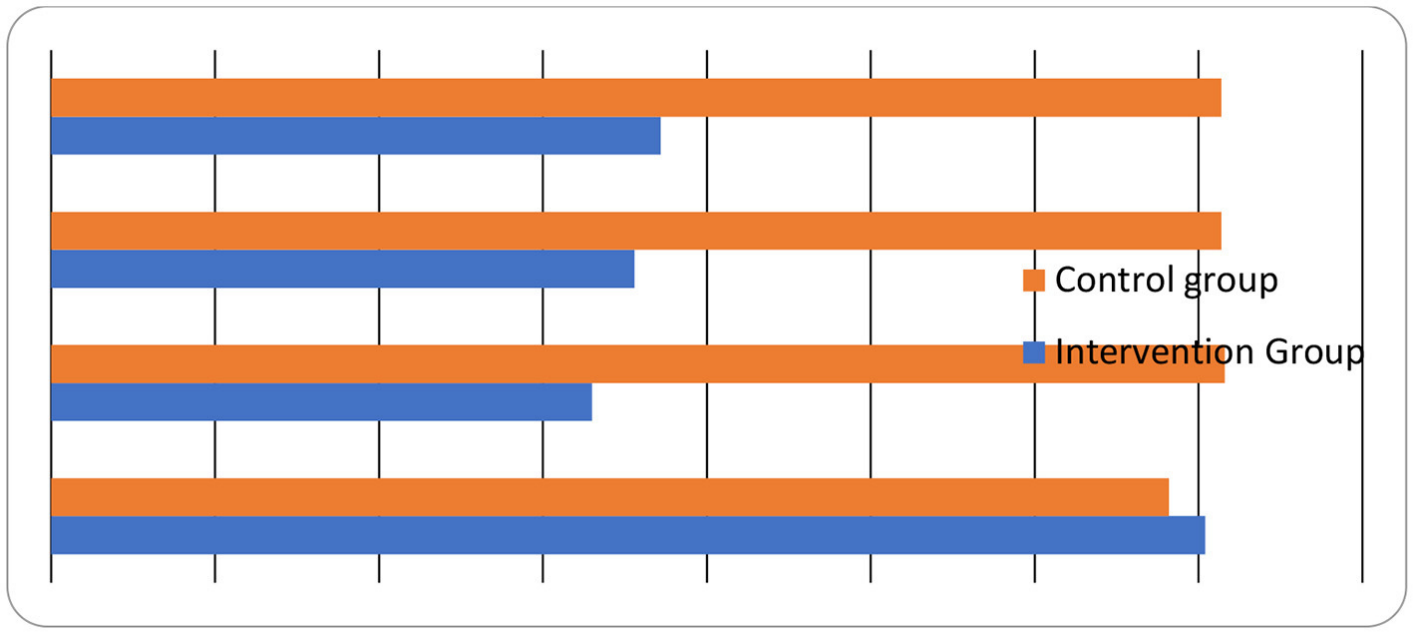
Main effect of time and intervention on participants' TSI scores.

Taking into account the measures obtained at Times 1, 2, 3, and 4, the within-subject effect analysis in Table 5 shows a significant effect of time on the TSI scores of the o-CBT group (*p* < 0.001, η^2^_*p*_ = 0.46). The partial eta square indicates a relatively high effect size. This indicates that the mean scores of the o-CBI group varied significantly across Times 1, 2, 3, and 4. Further, the Time X Group interaction effect indicated significant values, with a high effect size (*p* < 0.001, η^2^_*p*_ = 0.56**)** (also see Figure 3). This means that the difference in the group's TSI scores across time was due to the o-CBI intervention and not just because of the time difference.

**Table 5:** Repeated measure ANOVA for within-subject effects.

**Cases**	**Sum of squares**	**Df**	**Mean square**	**F**	**P**	**η^2^_p_**	**ω^2^**
Time	40.47	3	13.49	74.41	8.27 × 10^−35^	0.46	0.33
Time * Group	61.09	3	20.36	112.32	1.01 × 10^−46^	0.56	0.43
Residuals	47.32	261	0.18				

N, number in group; SD, standard deviation; Df, Degree of Freedom; p, probability value; η^2^_p_, partial eta square/effect size, ω^2^, Cohen' d effect size.

We also conducted a between-subject effect to ascertain the effects of the group on the mean TSI scores of the participants (Table 6). The data presented in Table 6 show that there was a significant difference in the participants' TSI mean scores based on the group (2.95 × 10^−33^), with a very high effect size, as indicated by the high value of partial eta square (η^2^_*p*_ = 0.81). This suggests that the o-CBI led to a significant difference in the TSI score compared to the control group.

**Table 6:** Repeated measure ANOVA for between-subject effects.

**Cases**	**Sum of squares**	**Df**	**Mean square**	**F**	**P**	**η^2^_p_**	**ω^2^**
Group	136.00	1	136.00	374.08	2.95 × 10^−33^	0.81	0.68
Residuals	31.63	87	0.36				

Type III sum of squares. N, number in group; SD, standard deviation; Df, Degree of Freedom; p, probability value; η^2^_p_, partial eta square/effect size, ω^2^, Cohen' d effect size.

The *post-hoc* analysis presented in Table 7 shows that the o-CBI group varied significantly in their TSI scores from Time 1 to 2 (mean difference = 1.78; *p* < 0.001). This indicates that there was a reduction of 1.78 in stress levels following the o-CBI. Further, there were non-significant changes in the TSI scores of the o-CBI group from Time 2 to 3 (mean difference = −0.05; *p* > 0.001) and from Time 3 to 4 (mean difference = −0.05; *p* < 0.001). This suggests that the reduction in the level of stress following the o-CBI was relatively stable and sustained; however, this outcome surprisingly showed that, though the improvement was sustained, it did not continue over time.

**Table 7:** *Post-hoc* analyses indicating the difference between groups and times for the global TSI scores.

		**95% CI for mean difference**					**95% CI for Cohen's d**			
		**Mean difference**	**Lower**	**Upper**	**SE**	**T**	**Cohen's d**	**Lower**	**Upper**	**p**_holm_
o-CBI, Time 1	o-CBI, Time 2	1.78	1.49	2.06	0.09	19.81	3.73	2.66	4.80	< 0.001
	o-CBI, Time 3	1.72	1.44	2.01	0.09	19.21	3.62	2.57	4.67	< 0.001
	o-CBI, Time 4	1.70	1.42	1.98	0.09	18.94	3.57	2.53	4.61	< 0.001
Control, Time 1	Control, Time 2	−0.18	−0.47	0.11	0.09	−2.00	−0.38	−0.99	0.23	0.61
	Control, Time 3	−0.18	−0.47	0.11	0.09	−1.98	−0.38	−0.98	0.23	0.61
	Control, Time 4	−0.17	−0.46	0.11	0.09	−1.91	−0.36	−0.97	0.24	0.61
o-CBI, Time 2	Control, Time 2	−1.76	−2.08	−1.44	0.10	−17.44	−3.70	−4.81	−2.59	< 0.001
	o-CBI, Time 3	−0.05	−0.34	0.23	0.09	−0.60	−0.11	−0.71	0.48	1.00
	Control, Time 3	−1.76	−2.08	−1.44	0.10	−17.43	−3.69	−4.80	−2.59	< 0.001
	o-CBI, Time 4	−0.08	−0.36	0.21	0.09	−0.87	−0.16	−0.76	0.43	1.00
	Control, Time 4	−1.75	−2.07	−1.44	0.10	−17.36	−3.68	−4.79	−2.58	< 0.001
Control, Time2	o-CBI, Time	1.71	1.39	2.03	0.10	16.91	3.59	2.50	4.67	< 0.001
	Control, Time 3	0.01	−0.28	0.29	0.09	0.02	3.94 × 10^−3^	−0.60	0.60	1.00
	o-CBI, Time 4	1.68	1.37	2.00	0.10	16.67	3.53	2.46	4.61	< 0.001
	Control, Time 4	0.00	−0.28	0.29	0.09	0.09	0.02	−0.58	0.62	1.00
o-CBI, Time 3	Control, Time 3	−1.71	−2.02	−1.39	0.10	−16.89	−3.58	−4.67	−2.50	< 0.001
	o-CBI, Time 4	−0.02	−0.31	0.26	0.09	−0.27	−0.05	−0.65	0.54	1.00
	Control, Time 4	−1.70	−2.02	−1.38	0.10	−16.83	−3.57	−4.65	−2.49	< 0.001
Control, Time 3	o-CBI, Time 4	1.68	1.36	2.00	0.10	16.65	3.53	2.45	4.61	< 0.001
	Control, Time 4	6.33 × 10^−3^	−0.28	0.29	0.09	0.07	0.01	−0.59	0.61	1.00
o-CBI, Time 4	Control, Time 4	−1.68	−1.99	−1.36	0.10	−16.59	−3.52	−4.59	−2.44	< 0.001

The data in Table 7 further show that the TSI scores of the control group did not change significantly over Time 1–2 (mean difference = −0.18; *p* > 0.001); Time 2–3 (mean difference = −0.01; *p* > 0.001); and Time 3–4 (mean difference = −0.00; *p* > 0.001). These indicate that the TSI scores of the control group were stable across times of evaluation, strengthening the idea that o-CBI was accountable for the changes that occurred in the intervention group. Figure 3 further demonstrates how TSI scores changed over time in the o-CBI and control groups. This outcome is presented in the bar chart (Figure 3).

**Figure 4:**
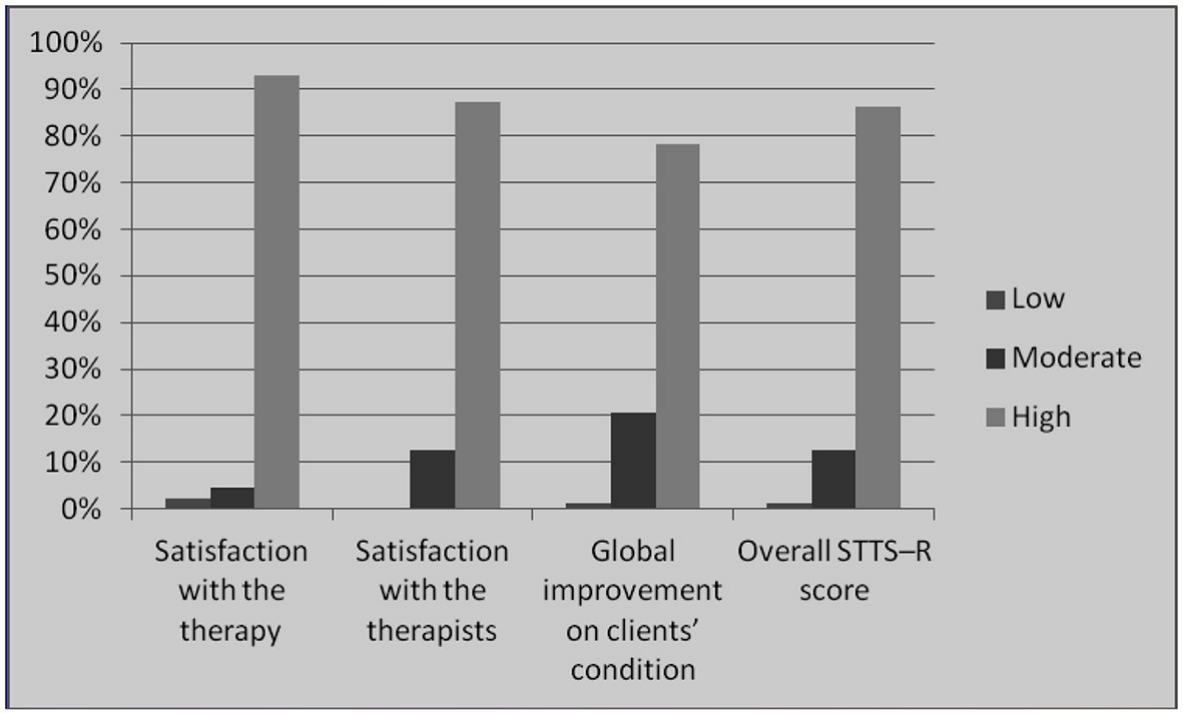
Bar chart showing participants' satisfaction with therapy.

To measure the participants' satisfaction with the o-CBI, we collected secondary data using the Satisfaction with Therapy and Therapist Scale-Revised (STTS-R). Table 5 and Figure 4 show the data gathered in this regard. The majority of the participants [81 (91.01%)] expressed high levels of satisfaction with the therapy they received. A total of 76 (85.39%) patients mentioned that they were very happy with their therapists, and 70 (78.65%) patients mentioned that the o-CBI helped them improve their overall health.

According to the participants' ratings in STTS–R (Table 8), 75 of the 89 participants who participated in the intervention (86.2%) were very satisfied with the o-CBI program. These results demonstrate the effectiveness of o-CBI in reducing teachers' levels of stress. Figure 4 shows more information about this outcome.

**Table 8:** Frequency and percentage of participants satisfaction with therapy.

**S/N**	**Item**	**Low**		**Moderate**		**High**	
		**F**	**%**	**F**	**%**	**F**	**%**
1	Satisfaction with the therapy	2	2.3	4	4.6	81	93.1
2	Satisfaction with the therapists	0	0	11	12.6	76	87.4
3	Global improvement in clients' condition	1	1.1	18	20.7	68	78.2
4	Overall STTS–R score	1	1.1	11	12.6	75	86.2

F, frequency.

## Discussion

The goal of this study was to confirm the efficacy of o-CBI in lowering job stress among ESL teachers. At baseline, the stress sources (SS) (t = 1.80, *p* = 0.993), stress manifestation (SM) (t = 2.28, *p* = 0.120), and total teachers' stress inventory (TTSI) scores (t = 2.16, *p* = 0.492) of the o-CBI and control groups did not differ substantially. When compared to the control group, the o-CBI resulted in a significant (*p* = 000) reduction in all dimensions of teachers' stress (SS, SM, and TTSI) at Time 2 (post-test), which was sustained through Time 3 (follow-up 1) and Time 4 (follow-up 2). The findings also demonstrated a significant interaction effect of time and intervention on the measures of participants' stress, suggesting that the decrease in the o-CBI group's stress levels over time was solely due to the o-CBI and not to the passage of time. While the stress levels of the participants in the control group did not alter significantly between baseline, post-intervention, and follow-up assessments, the o-CBI group saw a significant reduction in stress between baseline and post-treatment tests. This indicates that o-CBI alters participants' self-defeating cognition associated with work experience.

The significant reduction in ESL teachers' job stress following the o-CBI demonstrates that, even when objective working conditions remain the same, ESL teachers can change their perceptions regarding stressful experiences, resulting in reduced stress symptoms. The outcomes of this study strengthen other prior studies which show that CBI is effective for stress management. For instance, a number of studies have established the efficacy of cognitive behavioral interventions for stress reduction across the population (71, 75, 76, 88, 98). Further studies (74, 99) have shown that CBT-based stress management interventions are not only effective for stress but also for anxiety, hardiness, and self-efficacy.

A study by Leung et al. (67) used a brief cognitive-behavioral stress management program for secondary school teachers and found that the intervention effectively reduced stress. Though the result of Lueng and colleagues' study strengthens that of the present study, they used a face-to-face modality of CBI, while the present study intervention was delivered online. With increasing recommendations for the use of telemedicine and online treatment for mental health issues (100, 101), online cognitive behavioral intervention is being widely accepted for the treatment of stress and other mental disorders (93). In a preliminary study, Shirotsuki et al. (73) found internet-based cognitive behavior therapy effective in reducing stress among adults. Puertas-Gonzalez et al. (72) used online cognitive behavioral therapy as a psychological vaccine against stress during COVID-19. Kumar et al. (77) found internet-based cognitive behavioral therapy effective for treating psychiatric disorders. Other intervention studies have attested to the effectiveness of online CBI for stress (79, 102) and other mental health conditions (103).

Cognitive behavioral interventions are responsive to managing stress through three stress models: stimulus-based, response-based, and transaction-based models (40). CBI therapeutic modalities may be used to describe stress from all three perspectives, relieving the teachers. According to the stimulus-based model, stress arises when an objectively activating situation called a stressor occurs, whereas stress, according to the response-based model, occurs when emotional, physiological, and behavioral reactions to the stressor occur. Finally, transaction-based research suggests that stress is caused by negative subjective interpretations/cognitions and perspectives regarding a situation/stressor (A) (39, 41, 42).

The CBI modality lends itself to stress reduction by working on the transactional relationships across the three stress models, including stress as a stimulus, a reaction, and stress as a result of the stimulus (stressor) and response's transactional interactions (perceptions). Hence, CBI provides a framework for disputing negative perceptions of work experiences that lead to stress and has been a valuable approach to stress reduction and building employees' resources for stress management. With online CBI, participants tend to gain more expertise in stress management as they continue to employ these learned skills, and their perceptions of occupations become less stressful (104).

As a result, a previous study (105) found that a positive shift in stress perception can decrease physiological and psychological symptoms linked with job stress. Reduced job stress reduces psychopathological symptoms such as headaches, anxiety, and musculoskeletal difficulties, which can reduce employee effectiveness (106). As a result, reducing stress among ESL teachers may be a functional way to improve their health and classroom effectiveness (34). Improving the job effectiveness of ESL teachers translates to positive health and academic outcomes for the ESL students they teach. Online CBI is a low-cost program that improves the wellbeing and outcomes of instructors of ESL teachers and their students. This study has contributed to the existing pool of knowledge about the efficacy of o-CBI in a non-clinical group setting for stress reduction. It has helped to establish that group-based o-CBI is valuable for stress treatment among ESL teachers in Nigeria. This outcome can be verified in different locations.

The o-CBI intervention was shown to be well liked among participants in the study. Only a few studies have examined treatment preferences, expectations, usefulness, and satisfaction with mixed psychiatric treatments (107). Simon et al. (93) found internet-based CBT agreeable to participants based on a review of empirical studies. The therapy and study protocols were favorably regarded in other trials (93, 108). Participants in the o-CBI can access treatment materials and exercises from the comfort of their homes during online sessions and at their convenience, which reduces the cost of meetings (109). Furthermore, when compared to traditional face-to-face sessions, the o-CBI saves therapists' time.

### Study limitations and suggestions for future studies

This study is not without limitations. First, this study used a relatively small sample, which may limit the generalization of the study's results. Further study could apply a larger sample to confirm the effectiveness of the o-CBI. This study did not analyze data based on any mediating variable; future studies may fill this gap by considering mediating variables such as gender, experience, and level of education of the participants. Intention-to-treat or per-protocol analyses were not conducted; future studies may fill this gap. The o-CBI package could also be tested on different groups of employees who are suffering from chronic stress. Future research could compare the o-CBI and the traditional face-to-face CBI, as the current study did not look into this. Therapists who deal with teachers could consider using o-CBI to manage stress and provide the necessary intervention and support for ESL teachers. This study did not explore secondary outcome data such as depressive symptoms. Further studies can evaluate the presence of depressive symptoms that can result from the high levels of work-related stress among ESL teachers. Future studies could take similar studies further to evaluate depression in ESL teachers using Beck's Depression Inventory (BDI).

### Implications of the study for school health policy

The importance of this study for school health cannot be overestimated. The study findings have shown that online cognitive behavioral interventions can be useful for stress reduction in schools, especially for teachers. This may be relevant for the modification of mental health practices in schools in Nigeria and provides a theoretical basis for future policymaking with respect to physical and mental health policies in Nigerian schools. Stress is among the major health-threatening conditions experienced by both teachers and students in Nigeria (45, 110–113), and schools scarcely have or implement policies regarding stress management. Though the National Ministry of Education has set a comprehensive implementation plan for school safety through Education on Emergencies (114), there is still no policy regarding mental health crises such as stress in schools.

Currently, there is no regular framework to manage teachers' mental health in the Nigerian school system (115). According to Oshodi et al. (116), only 11.1% of school stakeholders had a form of school mental health service in their school; 75.8% confirmed the non-existence of mental health services, while 13.1% were unsure of the existence of mental health services. With the present study showing that o-CBI can effectively minimize stress, this can be built into school stress management policies for improved school health and increased academic performance. Techniques such as e-mental health can be adopted within the school policy system to cure the malaise of stress in Nigerian schools. This means that Nigerian public sector employees can benefit from routine stress management using o-CBI, which is cost-effective and evidence-based. Therefore, this study's outcome presents a viable implication for a new school health policy that will benefit both the teachers and the students. This is specifically important for those stressed about teaching English, which is not their local language.

## Conclusion

We conclude that the o-CBI is effective in reducing occupational stress among ESL teachers. The o-CBI for occupational stress was well received by the participants. The o-CBI for occupational stress was well received by the participants, showing high acceptability in the context.

## Data availability statement

The raw data supporting the conclusions of this article will be made available by the authors, without undue reservation.

## Ethics statement

The studies involving humans were approved by University of Nigeria Ethics Committee. The studies were conducted in accordance with the local legislation and institutional requirements. The participants provided their written informed consent to participate in this study.

## Author contributions

AE, VB, and BA conceptualized the topic and drew the initial draft. RN, JC, and LB designed the study. MA, ZO, and PM conducted the intervention. EA, ME, and VB validated the instruments, supervised the work, and participated in proofreading. FE, NA, and UE revised and proofread the manuscript. JN, NA, and SI collected and collated data. UE and LB analyzed the data and revised the manuscript. All authors contributed to the article and approved the submitted version.

## References

[1] Kim YK Ahn YS Kim K Yoon JH Roh J. Association between job stress and occupational injuries among Korean firefighters: a nationwide cross-sectional study. BMJ Open. (2016) 6:e012002. 10.1136/bmjopen-2016-012002 27888173 PMC5168540

[2] Khoshakhlagh AH Yazdanirad S Hatamnejad Y Khatooni E Kabir S Tajpoor A. The relations of job stress dimensions to safety climate and accidents occurrence among the workers. Heliyon. (2021) 7:e08082. 10.1016/j.heliyon.2021.e08082 34632148 PMC8488494

[3] Julià M Catalina-Romero C Calvo-Bonacho E Benavides FG. The impact of job stress due to the lack of organisational support on occupational injury. Occup Environ Med. (2013) 70:623–9. 10.1136/oemed-2012-101184 23716721

[4] Abdalla S Apramian SS Cantley LF Cullen MR Mock CN Nugent R Kobusingye O Smith KR. Occupation and risk for injuries. In: Injury Prevention and Environmental Health. 3rd, ed. The International Bank for Reconstruction and Development/The World Bank. (2017). 10.1596/978-1-4648-0522-6_ch6 30212110

[5] Ryan C Bergin M Chalder T Wells JS. Web-based interventions for the management of stress in the workplace: Focus, form, and efficacy. J Occup Health. (2017) 16:0227. 10.1539/joh.16-0227-RA 28320977 PMC5478505

[6] Mahfar M Senin AA. Managing stress at workplace using the Rational-Emotive Behavioral Therapy (REBT) approach. In: International Conference on Human Resource Development (2013).

[7] Leung MY Liang Q Olomolaiye P. Impact of job stressors and stress on the safety behavior and accidents of construction workers. J Manag Eng. (2016) 32:04015019. 10.1061/(ASCE)ME.1943-5479.0000373

[8] Luan R Pu W Dai L Yang R Wang P. Comparison of psychological stress levels and associated factors among healthcare workers, frontline workers, and the general public during the novel coronavirus pandemic. Front Psychiatry. (2020) 11:583971. 10.3389/fpsyt.2020.583971 33335490 PMC7736032

[9] Sumathy V Sudha G. Teachers' stress and type of school: Is there link? Res Expo Int Multidiscipl Res J. (2013) 11:23–30.

[10] Cappe E Bolduc M Poirier N Popa-Roch MA Boujut E. Teaching students with Autism Spectrum Disorder across various educational settings: The factors involved in burnout. Teach Teach Educ. (2017) 67:498–508. 10.1016/j.tate.2017.07.014

[11] AbuMadini MS Sakthivel M. A comparative study to determine the occupational stress level and professional burnout in special school teachers working in private and government schools. Glob J Health Sci. (2018) 10:42–53. 10.5539/gjhs.v10n3p42

[12] WHO. (2020). Occupational health: Stress at the workplace. Available online at: https://www.who.int/news-room/questions-and-answers/item/ccupational-health-stress-at-the-workplace (accessed December 7, 2022).

[13] Yusuf FA Olufunke YR Valentine MD. Causes and impact of stress on teachers' productivity as expressed by primary school teachers in Nigeria. Creat Educ. (2015) 6:1937. 10.4236/ce.2015.618199

[14] Herman KC Hickmon-Rosa JE Reinke WM. Empirically derived profiles of teacher stress, burnout, self-efficacy, and coping and associated student outcomes. J Posit Behav Interv. (2018) 20:90–100. 10.1177/1098300717732066

[15] Lambert R Boyle L Fitchett P McCarthy C. Risk for occupational stress among US kindergarten teachers. J Appl Dev Psychol. (2019) 61:13–20. 10.1016/j.appdev.2018.07.003

[16] MacIntyre PD Ross J Talbot K Mercer S Gregersen T Banga CA. Stressors, personality and wellbeing among language teachers. System. (2019) 82:26–38. 10.1016/j.system.2019.02.013

[17] Nayernia A Babayan Z EFL. teacher burnout and self-assessed language proficiency: exploring possible relationships. Lang Testing Asia. (2019) 9:1–16. 10.1186/s40468-019-0079-6

[18] Liu S Onwuegbuzie AJ. Chinese teachers' work stress and their turnover intention. Int J Educ Res. (2012) 53:160–170. 10.1016/j.ijer.2012.03.006

[19] Chang H. Stress and burnout in EFL teachers: the mediator role of self-efficacy. Frontiers in Psychology. (2022) 13:880281. 10.3389/fpsyg.2022.880281 35572332 PMC9096871

[20] Wettstein A Schneider S Grosse Holtforth M La Marca R. Teacher stress: A psychobiological approach to stressful interactions in the classroom. Front Educ. (2021) 6:681258. 10.3389/feduc.2021.681258

[21] Nnyigide N Anyaegbu O. Teaching and learning English in a second language situation: The case of some Igbo Teachers and students. New J African Stud. (2020) 16:160–75. 10.4314/og.v16i1.10

[22] Alqarni NA. Well-being and the Perception of Stress among EFL University Teachers in Saudi Arabia. J Lang Educ. (2021) 7:8–22. 10.17323/jle.2021.11494

[23] Yang HM. English as a foreign language teachers' well-being, their apprehension, and stress: the mediating role of hope and optimism. Front Psychol. (2022) 13:855282. 10.3389/fpsyg.2022.855282 35369148 PMC8965603

[24] Eun B Heining-Boynton AL. Impact of an English-as-a-second-language professional development program. J Educ Res. (2007) 101:36–49. 10.3200/JOER.101.1.36-49

[25] Haufiku I Mashebe P Abah J. Teaching challenges of english second language teachers in senior secondary schools in the ohangwena region, Namibia. Creat Educ. (2022) 13:1941–64. 10.4236/ce.2022.136121

[26] Iwe NN Chikamadu CP. Teaching English in low resource-environments: problems and prospects. Specialusis Ugdymas. (2022) 1:1298–316. Available online at: http://www.sumc.lt/index.php/se/article/view/153/155 (accessed February 20, 2021).

[27] Sadeghi K Richards JC. Professional development among English language teachers: challenges and recommendations for practice. Heliyon. (2021) 7:e08053. 10.1016/j.heliyon.2021.e08053 34622060 PMC8479616

[28] Wong CY. “ESL teachers are looked down upon”: Understanding the lived experience of a first-year ESL teacher with a culturally and linguistically diverse background. J Educ Res Pract. (2022) 12:291–303. 10.5590/JERAP.2022.12.1.20

[29] Obiegbu IR. Reading errors in second language learners. SAGE Open. (2018) 8:2158244018792973. 10.1177/2158244018792973

[30] Obiakor MI Malu D. Problem of teaching and learning of English language as a second language in secondary schools in Ankpa Local government Area of Kogi State. Afrn J Educ Manage Teach Entrepr Stud. (2020) 1:76–88. Available online at: https://www.ajemates.org/index.php/ajemates/article/view/14 (accessed November 15, 2022).

[31] Bakare OD Owolabi OA Bamigboye OB Bankole OM. Factors affecting library use by academic staff and students of federal University of Agriculture, Abeokuta, Ogun State. PNLA Quarterly. (2013) 77:3. Available online at: https://d1wqtxts1xzle7.cloudfront.net/53378230/Factors_Affecting_Library_Use_by_Academic_Staff_and_Students_of_Federal_University_of_Agriculture-libre.pdf?1496509009=&response-content-disposition=inline%3B+filename%3DFactors_Affecting_Library_Use_by_Academi.pdf&Expires=1694394189&Signature=XQXff8npD9J2I7ghvDUYly9FU8QYgww3QMK8oEzFlD3cs80YY8WetjAA14nOp-7s697cRrXZMIJwQaylr6Z-WC6FY~~RJbgkWezrA3opdLkJzA82U6KgA~iKHcsRTy8khD8jH4Sk6oZaoAdBDc4-AzLRKDxtLiu7TBZA1pMn5IbDYyvmgyS188nfBqDxHUDbCUlkOMdHJYGxegf7GloprgckPCGGVFEejVXwIwRi7UdRLFOcIQb1hETfREI7mSxYOSPtABPTJRVcZJYTEuJgpGVu3EuvgwXIB0MhIEVureoLe8lCABxUwPuXpwYLBnr8HMi4ilJoGAbbVgIRNK1COQ__&Key-Pair-Id=APKAJLOHF5GGSLRBV4ZA

[32] Elujekwute EC Nyita RH Elujekwute LA. Occupational stress and teacher's job performance in secondary schools in Makurdi Education Zone of Benue State, Nigeria. Unpublished M. Ed Dissertation, Benue State University, Makurdi. (2015).

[33] Gitonga MK Ndagi JM. Influence of occupational stress on teachers' performance in public secondary schools in Nyeri County, Nyeri South Sub County Kenya. Int J Bus Manage Invent. (2016) 5:23–9. Available online at: https://www.ijbmi.org/papers/Vol(5)5/version-2/D050502023029.pdf (accesses September 9, 2023).

[34] Hamlett C. How Stress Affects Your Work Performance. (2016). Available online at: https://smallbusiness.chron.com/stress-affects-work-performance-18040.html (accessed November 14, 2016).

[35] Danish RQ Qaseem S Mehmood T Ali QM Ali HF Shahid R. Work related stressors and teachers' performance: evidence from college teachers working in Punjab. Eur Scient J. (2019) 1:158–73. 10.19044/esj.2019.v15n4p158

[36] Asaloei SI Wolomasi AK Werang BR. Work-Related stress and performance among primary school teachers. Int J Eval Res Educ. (2020) 9:352–8. 10.11591/ijere.v9i2.20335

[37] Ukonu IO Edeoga G. Job-related stress among public junior secondary school teachers in Abuja, Nigeria. Int J Human Resour Stud. (2019) 9:2162–3058. 10.5296/ijhrs.v9i1.13589

[38] MacIntyre P Mercer S Gregersen T Hay A. The role of hope in language teachers' changing stress, coping, and well-being. System. (2022) 109:102881. 10.1016/j.system.2022.102881

[39] Rao JV Chandraiah K. Occupational stress, mental health and coping among information technology professionals. Indian J Occup Environ Med. (2012) 16:22. 10.4103/0019-5278.99686 23112503 PMC3482704

[40] Papathanasiou IV Tsaras K Neroliatsiou A Roupa A. Stress: Concepts, theoretical models and nursing interventions. Am J Nurs Sci. (2015) 4:45–50. 10.11648/j.ajns.s.2015040201.19

[41] Abrams M Ellis A. Rational emotive behaviour therapy in the treatment of stress. Br J Guid Counsell. (1994) 22:39–50. 10.1080/03069889408253664

[42] Quick JC Henderson DF. Occupational stress: Preventing suffering, enhancing wellbeing. Int J Environ Res Public Health. (2016) 13:459. 10.3390/ijerph13050459 27136575 PMC4881084

[43] Manabete SS John CA Makinde AA Duwa ST. Job stress among school administrators and teachers in Nigerian secondary schools and technical colleges. Int J Educ Learn Dev. (2016) 4:1–9. Available online at: http://www.eajournals.org/wp-content/uploads/Job-Stress-among-School-Administrators-and-Teachers-in-Nigerian-Secondary-Schools-and-Technical-Colleges.pdf (accessed September 5, 2022).

[44] Chadha M Sood K Malhotra S. Effects of organizational stress on quality of life of primary and secondary school teachers. Delhi Psychiat J. (2012) 15:342–6. Available online at: https://imsear.searo.who.int/handle/123456789/159671 (accessed September 7, 2022).

[45] Nwimo IO Onwunaka C. Stress among secondary school teachers in Ebonyi state, Nigeria: suggested interventions in the worksite Milieu. J Educ pract. (2015) 6:93–100. Available Online at: https://files.eric.ed.gov/fulltext/EJ1077456.pdf (accessed September 20, 2022).

[46] Okwaraji FE Aguwa EN. Burnout, psychological distress and job satisfaction among secondary school teachers in Enugu, South East Nigeria. Afr J Psychiat. (2015) 18:237–45. 10.4172/Psychiatry.1000198

[47] Manjula CMA. A study on personality factors causing stress among secondary school teachers, strength for today and bright hope for tomorrow. (2012) 12.

[48] Boujut E Popa-Roch M Palomares EA Dean A Cappe E. Self-efficacy and burnout in teachers of students with autism spectrum disorder. Res Autism Spectr Disord. (2017) 36:8–20. 10.1016/j.rasd.2017.01.002

[49] Yang L Zhao Y Wang Y Liu L Zhang X Li B. The effects of psychological stress on depression. Curr Neuropharmacol. (2015) 13:494–504. 10.2174/1570159X1304150831150507 26412069 PMC4790405

[50] Zarafshan H Mohammadi MR Ahmadi F Arsalani A. Job burnout among Iranian elementary school teachers of students with autism: A comparative study. Iran J Psychiatry. (2013) 8:20. Available online at: https://www.ncbi.nlm.nih.gov/pmc/articles/PMC3655226/pdf/IJPS-8-20.pdf (accessed September 13, 2022). 23682248 PMC3655226

[51] Atiyat OK. The level of psychological burnout at the teachers of students with autism disorders in light of a number of variables in Al-Riyadh Area. J Educ Learn. (2017) 6:159–74. 10.5539/jel.v6n4p159

[52] Hagaman JL Casey KJ. Teacher attrition in special education: Perspectives from the field. Teach Educ Special Educ. (2018) 41:277–91. 10.1177/0888406417725797

[53] Arun P Garg R Chavan BS. Stress and suicidal ideation among adolescents having academic difficulty. Ind Psychiatry J. (2017) 26:64. 10.4103/ipj.ipj_5_17 29456324 PMC5810170

[54] Malik NA Björkqvist K. Occupational stress and mental and musculoskeletal health among university teachers. Eur J Med Invest. (2018) 2:139–47. 10.14744/ejmi.2018.41636

[55] Ama JA. Challenges encountered by Learners of English as a second language. Available online at: https://owlcationcom/academia/amaado. (2018).

[56] Emenanjo EN. Language and the national policy on education. In: Actes de La 8ème Conference Bienniale de La Modern Languages Association of Nigeria (MLAN) Tenue à. (1991).

[57] Ibrahim J Gwandu SA. Language policy on education in Nigeria: Challenges of multilingual education and future of English language. Am Res J English Liter. (2016) 2:1–10.

[58] Agnes LM Thembekile MN Sive M. The improvement of learners' proficiency in English second language through remedial work. Arabic Lang Liter Cult. (2021) 6:1. 10.11648/j.allc.20210601.11

[59] Okebukola PA Owolabi O Okebukola FO. Mother tongue as default language of instruction in lower primary science classes: Tension between policy prescription and practice in Nigeria. J Res Sci Teach. (2013) 50:62–81. 10.1002/tea.21070

[60] Cabardo JR. Reading proficiency level of students: Basis for reading intervention program. (2015). Available online at: https://www.researchgate.net/profile/Jimmy-Rey-Cabardo/publication/314602653_Reading_Proficiency_Level_of_Students_Basis_for_Reading_Intervention_Program/links/5c525da8299bf12be3efe630/Reading-Proficiency-Level-of-Students-Basis-for-Reading-Intervention-Program.pdf (accessed January 22, 2023).

[61] Oliver R Young S. Improving reading fluency and comprehension in adult ESL learners using bottom-up and top-down vocabulary training. Stud Second Lang Learn Teach. (2016) 6:111–33. 10.14746/ssllt.2016.6.1.6

[62] Nyarko K Kugbey N Kofi CC Cole YA Adentwi KI. English reading proficiency and academic performance among lower primary school children in Ghana. SAGE Open. (2018) 8:2158244018797019. 10.1177/2158244018797019

[63] Vyomakesisri TIPPABHOTLA. Challenges in learning English as secondary language. Int J English Literat. (2017) 7:21–4. 10.24247/ijeldec20174

[64] Lambert C Zhang G. Engagement in the use of English and Chinese as foreign languages: the role of learner-generated content in instructional task design. Modern Lang J. (2019) 103:391–11. 10.1111/modl.12560

[65] Beck AT. Cognitive Therapy and Emotional Disorders. New York: New American library. (1976).

[66] Fenn K Byrne M. The key principles of cognitive behavioural therapy. InnovAiT. (2013) 6:579–85. 10.1177/1755738012471029

[67] Leung SS Chiang VC Chui YY Mak YW Wong DF. A brief cognitive-behavioral stress management program for secondary school teachers. J Occup Health. (2011) 53:23–35. 10.1539/joh.L10037 21079374

[68] Svärdman F Sjöwall D Lindsäter E. Internet-delivered cognitive behavioral interventions to reduce elevated stress: A systematic review and meta-analysis. Internet Interv. (2022) 10:553. 10.31234/osf.io/e7cxd 35781929 PMC9240371

[69] Ulaş S Seçer I. Developing a cbt-based intervention program for reducing school burnout and investigating its effectiveness with mixed methods research. Front Psychol. (2022) 13:884912. 10.3389/fpsyg.2022.884912 35923738 PMC9342602

[70] Heber E Ebert DD Lehr D Cuijpers P Berking M Nobis S. The benefit of web-and computer-based interventions for stress: a systematic review and meta-analysis. J Med Intern Res. (2017) 19:e32. 10.2196/jmir.5774 28213341 PMC5336602

[71] Li J Li X Jiang J Xu X Wu J Xu Y. The effect of cognitive behavioral therapy on depression, anxiety, and stress in patients with COVID-19: a randomized controlled trial. Front Psychiat. (2020) 11:580827. 10.3389/fpsyt.2020.580827 33192723 PMC7661854

[72] Puertas-Gonzalez JA Marino-Narvaez C Romero-Gonzalez B Sanchez-Perez GM Peralta-Ramirez MI. Online cognitive behavioural therapy as a psychological vaccine against stress during the COVID-19 pandemic in pregnant women: A randomised controlled trial. J Psychiatr Res. (2022) 152:397–405. 10.1016/j.jpsychires.2022.07.016 35830754 PMC9259661

[73] Shirotsuki K Uehara S Adachi S Nakao M. Internet-based cognitive behavior therapy for stress and anxiety among young japanese adults: A preliminary study. Psych. (2019) 1:353–63. 10.3390/psych1010025

[74] Sahranavard S Esmaeili A Salehiniya H Behdani S. The effectiveness of group training of cognitive behavioral therapy-based stress management on anxiety, hardiness and self-efficacy in female medical students. J Educ Health Promot. (2019) 8:49. 10.1051/bmdcn/2018080423 30993142 PMC6432834

[75] Onyishi CN Ede MO Ossai OV Ugwuanyi CS. Rational emotive occupational health coaching in the management of police subjective well-being and work ability: A case of repeated measures. J Police Crim Psychol. (2021) 36:96–111. 10.1007/s11896-019-09357-y

[76] Okeke FC Onyishi CN Nwankwor PP Ekwueme SC A. blended rational emotive occupational health coaching for job-stress among teachers of children with special education needs. Internet Interv. (2021) 26:100482. 10.1016/j.invent.2021.100482 34824983 PMC8604685

[77] Kumar V Sattar Y Bseiso A Khan S Rutkofsky IH. The effectiveness of internet-based cognitive behavioral therapy in treatment of psychiatric disorders. Cureus. (2017) 9:e1626. 10.7759/cureus.1626 29098136 PMC5659300

[78] World Health Organization. Recommendations on digital interventions for health system strengthening: WHO guideline. In: Recommendations on digital interventions for health system strengthening: WHO guideline. (2019). p. 150. 31162915

[79] Weiner L Berna F Nourry N Severac F Vidailhet P Mengin AC. Efficacy of an online cognitive behavioral therapy program developed for healthcare workers during the COVID-19 pandemic: the REduction of STress (REST) study protocol for a randomized controlled trial. Trials. (2020) 21:1–10. 10.1186/s13063-020-04772-7 33087178 PMC7576984

[80] Romijn G Riper H Kok R Donker T Goorden M van Roijen LH. Cost-effectiveness of blended vs. face-to-face cognitive behavioural therapy for severe anxiety disorders: study protocol of a randomized controlled trial. BMC Psychiat. (2015) 15:1–10. 10.1186/s12888-015-0697-1 26651478 PMC4676824

[81] Carroll KM Rounsaville BJ. Computer-assisted therapy in psychiatry: Be brave—it'sa new world. Curr Psychiatry Rep. (2010) 12:426–32. 10.1007/s11920-010-0146-2 20683681 PMC2967758

[82] Ong CW Terry CL Levin ME Twohig MP. Examining the feasibility and effectiveness of online acceptance and commitment therapy self-help in a quasi-stepped care model: A pilot study. Psychol Serv. (2023) 20:1–36. 10.1037/ser0000596 34735201

[83] World Medical Association. General Assembly of the World Medical Association Declaration of Helsinki: ethical principles for medical research involving human subjects. J Am College Dent. (2014) 81:14. Available online at: https://pubmed.ncbi.nlm.nih.gov/25951678/ (accessed January 3, 2021). 25951678

[84] APA. Report Highlights: Stress in America: Paying for our health. Technical Report. (2017).

[85] Fredriksson-Larsson U Brink E Grankvist G Jonsdottir IH Alsen P. The single-item measure of stress symptoms after myocardial infarction and its association with fatigue. Open J Nurs. (2015) 5:345. 10.4236/ojn.2015.54037

[86] Moldovan CP. AM Happy Scale: Reliability and Validity of a Single-Item Measure of Happiness. Loma Linda University Electronic Theses, Dissertations and Projects (2017). p. 438.

[87] Fimian MJ. The development of an instrument to measure occupational stress in teachers: The Teacher Stress Inventory. J Occupat Psychol. (1984) 57:277–93. 10.1111/j.2044-8325.1984.tb00169.x

[88] Ogba FN Onyishi CN Victor-Aigbodion V Abada IM Eze UN Obiweluozo PE. Managing job stress in teachers of children with autism: A rational emotive occupational health coaching control trial. Medicine. (2020) 99:e21651. 10.1097/MD.0000000000021651 32898998 PMC7478671

[89] Fimian MJ Fastenau PS. The validity and reliability of the Teacher Stress Inventory: A re-analysis of aggregate data. J Organ Behav. (1990) 11:151–7. 10.1002/job.4030110206

[90] Boshoff SM. Validation of the Teacher Stress Inventory (TSI) in a South African context: the SABPA study. Doctoral dissertation, North-West University. (2011).

[91] Oei TPS Shuttlewood GJ. Development of a satisfaction with therapy and therapist scale. Austr New Zeal J Psychiat. (1999) 33:748–53. 10.1080/j.1440-1614.1999.00628.x 10545001

[92] Sidani S Epstein DR Fox M. Psychometric evaluation of a multi-dimensional measure of satisfaction with behavioral interventions. Res Nurs Health. (2017) 40:459–69. 10.1002/nur.21808 28857205 PMC5657530

[93] Simon N McGillivray L Roberts NP Barawi K Lewis CE Bisson JI. Acceptability of internet-based cognitive behavioural therapy (i-CBT) for post-traumatic stress disorder (PTSD): a systematic review. Eur J Psychotraumatol. (2019) 10:1646092. 10.1080/20008198.2019.1646092 31497259 PMC6719262

[94] Demidova I. Treating Adult Women With Depression Through Videoconferencing. (2017). Available online at: https://scholarworks.waldenu.edu/cgi/viewcontent.cgi?article=4447&context=dissertations (accessed December 3, 2022).

[95] Ellis A. Changing rational-emotive therapy (RET) to rational emotive behavior therapy (REBT). J Ration Emot Cogn Behav Ther. (1996) 13:85–9. 10.1007/BF02354453

[96] Boß L Angerer P Dragano N Ebert D Engels M Heber E. Comparative effectiveness of guided internet-based stress management training versus established in-person group training in employees–study protocol for a pragmatic, randomized, non-inferiority trial. BMC Public Health. (2021) 21:1–13. 10.1186/s12889-021-12229-y 34837999 PMC8626923

[97] Desveaux L Agarwal P Shaw J Hensel JM Mukerji G Onabajo N. A randomized wait-list control trial to evaluate the impact of a mobile application to improve self-management of individuals with type 2 diabetes: a study protocol. BMC Med Inform Decis Mak. (2016) 16:1–11. 10.1186/s12911-016-0381-5 27842539 PMC5109669

[98] Obiweluozo PE Dike IC Ogba FN Elom CO Orabueze FO Okoye-Ugwu S. Stress in teachers of children with neuro-developmental disorders: Effect of blended rational emotive behavioral therapy. Sci Prog. (2021) 104:00368504211050278. 10.1177/00368504211050278 34783626 PMC10402289

[99] Akanaeme IN Ekwealor FN Ifeluni CN Onyishi CN Obikwelu CL Ohia NC. Managing job stress among teachers of children with autism spectrum disorders: A randomized controlled trial of cognitive behavioral therapy with yoga. Medicine. (2021) 100:e27312. 10.1097/MD.0000000000027312 34797272 PMC8601364

[100] Andersson G Titov N. Advantages and limitations of Internet-based interventions for common mental disorders. World Psychiat. (2014) 13:4–11. 10.1002/wps.20083 24497236 PMC3918007

[101] Banbury A Nancarrow S Dart J Gray L Parkinson L. Telehealth interventions delivering home-based support group videoconferencing: systematic review. J Med Internet Res. (2018) 20:e8090. 10.2196/jmir.8090 PMC5816261 29396387

[102] Lindsäter E Axelsson E Salomonsson S Santoft F Ejeby K Ljotsson B. Internet-based cognitive behavioral therapy for chronic stress: a randomized controlled trial. Psychother Psychosom. (2018) 87:296–305. 10.1159/000490742 30041167

[103] Hedman E Andersson G Ljótsson B Andersson E Rück C Mörtberg E. Internet-based cognitive behavior therapy vs. cognitive behavioral group therapy for social anxiety disorder: a randomized controlled non-inferiority trial. PLoS ONE. (2011) 6:e18001. 10.1371/journal.pone.0018001 21483704 PMC3070741

[104] Thomas G. How to do Your Research Project: A Guide for Students. London: Sage. (2017).

[105] Suleman Q Hussain I Shehzad S Syed MA Raja SA. Relationship between perceived occupational stress and psychological well-being among secondary school heads in Khyber Pakhtunkhwa, Pakistan. PLoS ONE. (2018) 13:e0208143. 10.1371/journal.pone.0208143 30540807 PMC6291082

[106] Igu NC Onyishi CN Amujiri BA Binuomote MO Modebelu MN Okafor IP. Raising leadership self-efficacy and minimizing organizational burnout among school administrators in a GROW model of cognitive behavioral coaching. J Lead Organ Stud. (2023). 10.1177/15480518231171748

[107] Campos D Mira A Bretón-López J Castilla D Botella C Baños RM. The acceptability of an internet-based exposure treatment for flying phobia with and without therapist guidance: patients' expectations, satisfaction, treatment preferences, and usability. Neuropsychiatr Dis Treat. (2018) 14:879–92. 10.2147/NDT.S153041 29636613 PMC5880416

[108] Mackie C Dunn N MacLean S Testa V Heisel M Hatcher S. qualitative study of a blended therapy using problem solving therapy with a customised smartphone app in men who present to hospital with intentional self-harm. BMJ Ment Health. (2017) 20:118–22. 10.1136/eb-2017-102764 29030503 PMC10516399

[109] Hedman E Ljotsson B Kaldo V Hesser H El Alaoui S Kraepelien M. Effectiveness of Internet-based cognitive behaviour therapy for depression in routine psychiatric care. J Affect Disord. (2014) 155:49–58. 10.1016/j.jad.2013.10.023 24238951

[110] Nakpodia ED. Rating of stress factors in delta state secondary schools, Nigeria. Int J Dev Manage Rev. (2012) 7:9285.

[111] Akuezuilo JA Azuji IM. Extent of school-related stress occurrence among secondary school teachers in Anambra State: Implication for health counselling. Int J Res Sci Innov. (2019) 6:90–4.

[112] Subair ST Oluwaseun AO Aliyu MO. Job stress and teachers' coping strategies in nigerian schools. Am J Soc Sci Human. (2021) 6:1–13. 10.20448/801.61.1.13

[113] Tsagem SY Lema MM. Occupational stress and burnout among secondary school teachers in Sokoto metropolis–Nigeria: how does coping strategies matter? Int J Soc Sci Hum Res. (2022) 5:2889. 10.47191/ijsshr/v5-i7-16

[114] National Policy on Safety Security Violence-Free Schools in Nigeria. National policy on safety, security and violence-free schools with its implementing guidelines. Available online at: https://education.gov.ng/wp-content/uploads/2021/09/National-Policy-on-SSVFSN.pdf (2021).

[115] Ryan GK Nwefoh E Aguocha C Ode PO Okpoju SO Ocheche P. Partnership for the implementation of mental health policy in Nigeria: a case study of the Comprehensive Community Mental Health Programme in Benue State. Int J Ment Health Syst. (2020) 14:1–13. 10.1186/s13033-020-00344-z 32110245 PMC7033947

[116] Oshodi OY Aina OF Adeyemi JD Oduguwa TO Ogundipe OA. Perceived needs for school mental health among stakeholders in districts of South-west Nigeria. J Public Health Epidemiol. (2013) 5:153–9. 10.5897/JPHE12.084

